# Depression, anxiety, stress, and PTSD symptoms during the first and second COVID-19 waves: a comparison of elderly, middle-aged, and young people in Iran

**DOI:** 10.1186/s12888-023-04677-0

**Published:** 2023-03-23

**Authors:** Hajar Pasha, Shabnam Omidvar, Mahbobeh Faramarzi, Afsaneh Bakhtiari

**Affiliations:** Social Determinants of Health Research Center, Health Research Institute, Babol University of Medical Sciences, Babol, Iran

**Keywords:** COVID-19, Age groups, Anxiety, Depression, Psychological stress, Post-traumatic stress disorder

## Abstract

**Background:**

A widespread outbreak of epidemics like Covid-19 is a lethal threat to physical and mental health. Recent studies reported a higher prevalence of mental problems in younger individuals, contrary to the general assumption expected in older people. Therefore, it is necessary to compare anxiety, stress, depression and PTSD (post-traumatic stress disorder) symptoms in different age groups during the Covid-19 crisis.

**Methods:**

A cross-sectional online survey was performed (from Dec. 2020 to Feb. 2021) on three age groups of elderly, middle-aged and young people. Data were collected by DASS-21 (Depression, Anxiety and Stress Scale) and IES-R (Impact of Event Revised Scale) and analyzed using ANOVA, χ2 test and logistic regression analysis.

**Results:**

Overall, 601 participants completed the questionnaires, including 23.3% of the elderly (≥ 60 years), 29.5% of the young (18–29 years) and 47.3% of the middle-aged (30–59 years) with 71.4% of women. The logistic regression analysis revealed that the risk of PTSD in young people was higher than in the elderly (β = 2.242, CI: 1.03–4.87, *P* = 0.041), while the risk of depression, anxiety and stress did not differ significantly among the three age groups. Female gender, occupation, lower economic status, solitary life, and chronic disease were risk factors for psychological symptoms during the Covid-19 pandemic.

**Conclusion:**

Findings on the higher odds ratio of PTSD symptoms in younger individuals have interestingly potential implications to meet the needs of mental health services during Covid-19.

**Supplementary Information:**

The online version contains supplementary material available at 10.1186/s12888-023-04677-0.

## Introduction

The widespread occurrence of pandemics, such as the Covid-19, is closely related to the symptoms of mental health disorders and psychiatric diseases, regardless of infection [1]. Past studies on infectious diseases have shown a larger number of people with mental disorders caused by the pandemics than the physical patients [2]. The detrimental effects of the Covid-19 on psychological health are deeper and broader than previous pandemics, hence the expectation of more adverse psychological consequences. Studies have shown that the Covid-19-induced stress was 1.5 times greater than that of MERS and 1.4 times greater than local earthquakes [3], which indicate the great impact of this disease on physiological and psychological well-being, as well as urgent measures for the psychological health of people in a community [5, 6].

The impact of the pandemic on mental health, including high rates of anxiety, depression, and post-traumatic stress disorder (PTSD) in the general population, has been reported in several countries across the Americas, Asia, the Middle East, and Europe [4], with few studies on specific age groups such as young adults and the elderly [7, 8]. Therefore, there is a need for studies that simultaneously compare age groups in terms of psychological responses, especially in low-income countries with more serious crises during pandemics [5].

Contraction with Covid-19 and the mortality associated with it occur more in people of higher ages [1, 6], but recent meta-analyses have shown that during the pandemic, levels of anxiety, depression, and stress were significantly higher in the 21–40 age group [4, 7, 8]. Hung et al. reported the overall prevalence of anxiety and depression in the community as 35.1% and 20.1%, which was significantly higher in younger people [9]. In addition, a study on psychological distress in the general population of Canada found that people aged 18–30 or over 60 had the highest scores on the Covid-19 Peritraumatic Distress Index [10]. Several smaller-scale studies (*n* < 400) have also demonstrated worsening psychological symptoms in groups of young people in the USA, Italy, India, Switzerland, and China [11]. One study on individuals aged 55 years or over also showed an increase in mild depression and anxiety symptoms, but no change in the frequency of moderate symptoms. Compared to young adults, older adults showed less anxiety and depression, and their mental health remained stable despite exacerbated loneliness during the pandemic [12]. However, some studies reported more emotional responses [13] and psychosis [14] in the elderly compared to other age groups during the Covid-19 crisis.

The health of the elderly is emphasized during the Covid-19 outbreak because they have a higher prevalence of pre-existing mental disorders and comorbidities, as well as the longest period of social isolation, which develops or worsens mental disorders. In addition, the Covid-19 trauma causes detestation and bias towards the patients or certain groups such as healthcare workers and the elderly [14]. Recently, the development of hatred and discrimination against vulnerable elderly people against Covid-19 has been raised as a major social concern [15]. On the other hand, some studies suggested that older age may buffer against the COVID-19-related impact on mental health. However, senior citizens have varying adaptability to hardship depending on cultural, social, economic, and other factors. Taken together, the impact of Covid-19 is expected to vary across countries and older subpopulations. In addition, much of the literature on the vulnerability of older adults was based on anecdotal reports of prior experience with medical health crises and natural disasters [16].

Various factors are associated with the emergence of psychiatric symptoms in the Covid-19 crisis such as demographic factors, economic challenges, contact with social media, coping styles, quality of life, social support, psychological resilience, history of chronic diseases, and medical/psychiatric diseases, and government support policies [17–20].

Trying to maintain mental health during the covid-19 is as important as trying to prevent and treat it in all age groups considering the significant effects of the Covid-19 on the mental health of younger people in a meta-analysis [11]. Therefore, careful observations and further studies are necessary to find out the psychological effects of the pandemic on people at risk for preventive interventions. This study compares depression, anxiety, stress, and PTSD symptoms during the epidemic in the elderly, middle-aged, and young individuals. Some predictive factors have also been assessed.

## Methods

This cross-sectional study was conducted to assess the mental health and the impact of Covid-19 on the elderly compared to other age groups in Iran. Since the pandemic in Iran started on 19 February 2020, following its worldwide spread, the present study was conducted from December 2020 to February 2021 to investigate PTSD symptoms (after the first and second waves of 2020).

### Participants

The statistical population consisted of the elderly, middle-aged, and young individuals with eligibility for the study. According to the age guide of the Ministry of Health of Iran (integrated care plan), the age groups were as follows: young age group (18–29 years), middle-aged group (30–59) years and old age (60 and above). The inclusion criteria were people 18 years old and above with Iranian nationality and agreeing to participate in the study. The participants with an adverse condition in the previous three months were excluded from the study.

### Sampling and sample size

The sample size was estimated based on Green's law [21]. According to this law, the estimation of the minimum acceptable sample size in the regression technique is based on the variables of the predictors in the model. Considering that there were 8 predictor variables in the present study, an acceptable sample size with a response rate of 80%, 200 people in each group was considered.

### Data collection

Data collection tools included demographic characteristics questionnaire (age, gender, employment status, education, income adequacy from the individual perspective, marital status, chronic disease history, coexistence), Impact of Event Scale-Revised (IES-R) and Depression, Anxiety and Stress Scale (DASS).

Taking into account social distance, the survey was conducted online in all provinces of the country. The questionnaires were designed on Porsline and shared on social media such as Telegram, WhatsApp, LinkedIn, and Facebook. We also distributed the questionnaires on social media channels in all provinces and cities to increase the level of responsiveness. Eligible people completed the questionnaire by clicking on the link (https://Survey.Porsline.Ir/S/G5rrHca/). The survey started with the statement: "Participation in the study is completely voluntary and that the information you would share with the researchers would be strictly confidential." In this way, only those who were willing to participate in the research entered the questionnaire completion stage. After confirming the understanding of this issue with the participants, the average completion of the questionnaires lasted 5 min.

## Measurements

### Impact of Event Scale-Revised (IES-R)

The IES-R was designed to parallel the DSM-IV criteria for PTSD symptoms. The scale has 22 items and consists of three subscales comprising intrusion, avoidance, and hyperarousal. The response range is from 0 (no symptoms) to 4 (extremely high level of symptoms) on a Likert scale. The IES-R yields a total score (0 to 88) and subscale scores can also be calculated. A score ≥ 33 was considered the cut-off point on the IES-R to qualify as an indicator of PTSD symptoms in an adult or senior population [22]. A higher overall score indicates more impairment. Persian version of the scale has been shown to have high validity, test–retest reliability (*r* = 0.8–0.98, *P* < 0.001), and good internal consistency with Cronbach α between 0.67–0.87. The Cronbach α coefficients for the IES-R subscales were from 0.84 to 0.93 (22).

### Depression, Anxiety and Stress Scale (DASS)

The Depression, Anxiety and Stress Scale -21 Items (DASS-21) is a set of three self-report scales designed to measure the emotional states of depression, anxiety and stress over the past week. Each of the three DASS-21 scales contains seven items. Its response range is from 0 (never) to 3 (forever) on a Likert scale. Before interpreting the scores, the summed numbers in each subscale need to be multiplied by 2 (this is because the DASS 21 is the short form of the scale). DASS intensity is rated from normal to very severe in five categories. DASS scores of ≥ 28 for depression, ≥ 20 for anxiety, and ≥ 34 for stress are considered very severe. The Persian version of the questionnaire has a reasonable degree of internal consistency, convergent and concurrent validities with Cronbach α of 0.94. Test–retest reliability for depression, stress, and anxiety scales were 0.77, 0.85, and 0.89, respectively [23].

### Statistical analysis

Once data were collected, all questionnaires were then entered into a customized Excel-based system. All data were subsequently imported into and analyzed via SPSS v. 22.0 (SPSS Inc., Chicago, Illinois, USA). The assumption of normality of the data has been met (Supplementary Fig. 1A to Fig. 7c). Statistical analysis was performed using regression models. Simple and multiple logistic regressions in categorical variables were carried out to detect predictor factors in IES-R and DASS among the participants. An ANOVA and χ2 test were applied to compare mean (IES-R, DASS) and frequency of changes in demographic characteristics in groups, respectivly. *P* < 0.05 was statistically significant.

## Results

### Descriptive analysis

A total of 601 participants completed the questionnaires, including 23.3% of the elderly, 29.5% of the young and 47.3% of the middle-aged. Of these, 71.4% were women and 28.6% were men. The personal characteristics of the participants were shown in Table 1. The results of depression, anxiety and stress in the Covid-19 epidemic showed that 39% of the participants did not have depression, 11.8% had mild, 21.4% moderate, 13.4% sever, and 14.4%% very severe depression. The anxiety percentages were 5.1% mild, 23.7% moderate, 11.3% sever, 22.1% very sever and 37.8% normal condition. Furthermore, 38% of them did not have stress, 11.3% had mild, 19.9% moderate, 22.2% sever, and 8.6%% very severe stress. Less than half of the people (40.3%) had experienced high PTSD symptoms.

**Table 1:** Personal characteristics of the study population's age groups

Variable	All (*n* = 601) N (%)	Age groups		
		**Young**	**Middle-aged**	**Elderly**
**Gender**				
Female	429(71.4)	129(72.9)	221(77.8)	79(56.4)
Male	172(28.6)	48(27.1)	63(22.2)	61(43.6)
**Occupation**				
Health care workers	79(13.1)	14(7.9)	57(20.1)	8(5.7)
non-healthcare workers	61(10.1)	4(2.3)	49(17.3)	8(5.7)
Student/teacher	194(32.3)	135(76.3)	47(16.5)	12(8.6)
Homemaker	144(24.0)	15(8.5)	88(31.0)	41(29.3)
Others	123(20.5)	9(5.1)	43(15.1)	71(50.7)
**Educational level**				
Literacy	23(3.8)	0(0)	1(0.4)	22(15.7)
< Diploma	46(7.7)	4(2.3)	11(3.9)	31(22.1)
Diploma	113(18.8)	23(13.0)	50(17.9)	40(28.6)
University	419(69.7)	150(84.7)	222(78.2)	47(33.6)
**Economic status (from the individual perspective)**				
Enough	309(51.4)	102(57.6)	156(54.9)	51(36.4)
Nearly enough	219(36.4)	66(37.3)	99(34.9)	54(38.6)
Not enough	73(12.1)	9(5.1)	29(10.2)	35(25.0)
**Marital status**				
Married	390(64.9)	44(24.9)	244(85.9)	102(72.9)
Single	211(35.1)	133(75.1)	40 (14.1)	38 (27.1)
**Underlying disease**				
No	449(74.7)	167(94.4)	214(75.4)	68(48.6)
Yes	152(25.3)	10(5.6)	70(24.6)	72(51.4)
**Coexistence**				
Alone	33(5.5)	3(1.7)	10(3.5)	20(14.3)
Living with family (parents/spouse and children)	533 (94.5)	168(96.6)	264(92.9)	101(72.2)
Living with others (children and friend)	35(5.8)	6(3.4)	10(3.6)	19(13.5)
**PTSD symptoms**				
High PTSD symptoms	243(40.3)	72(40.2)	112(39.4)	59(42.1)
Low PTSD symptoms	360(59.7)	106(59.8)	172(60.6)	81(57.9)
**DASS**				
Depression	368(61)	117(65.4)	163(57.4)	88(62.9)
Anxiety	375(62.2)	116(64.8)	165(58.1)	94(67.1)
Stress	374(62)	117(65.4)	175(61.6)	82(58.6)

### DASS and PTSD symptoms among the age groups

Comparison of depression, anxiety, stress and PTSD symptoms among the age groups showed a statistically significant difference in depression (*p* = 0.037), anxiety (*p* = 0.001), stress (*p* = 0.029) and hyperarousal subscale of PTSD (*p* = 0.035). Pairwise AVOVA revealed that young people had higher mean scores of depression than the middle-aged group. Also, elderly people had a higher mean score of anxiety, stress, and hyperarousal compared with the middle-aged group. There was no difference between young and elderly people regarding the mean scores of depression, stress, anxiety, and PTSD symptoms (Table 2).

**Table 2:** Comparison of mean of PTSD symptoms, depression, anxiety, and stress in the study population's age groups

**Variables**		**Age groups**			**F**	***p*-value**
	**Total** **(*n* = 601)**	**Young**	**Middle-aged**	**Elderly**		
**PTSD symptoms**						
Intrusion	9.29(7.07)	8.77(6.71)	9.15(7.04)	10.27(7.52)	1.888	0.152
Avoidance	12.23(7.21)	12.54(7.82)	11.89(6.93)	12.55(6.94)	0.612	0.543
Hyperarousal	6.96(5.60)	6.59(5.49)	6.66(5.58)	8.03(5.71)	3.385	0.035
Total score	28.49(17.34)	27.89(17.46)	27.70(16.86)	30.86(18.03)	1.704	0.183
**DASS**						
Depression	14.26(10.43)	15.78(10.60)	13.23(10.33)	14.4(10.26)	3.306	0.037
Anxiety	11.69(8.83)	12.18(8.95)	10.39(7.90)	13.68(9.91)	7.043	0.001
Stress	19.93(9.47)	20.28(9.38)	18.94(9.98)	21.48(8.29)	3.579	0.029

The values are mean (SD)

### DASS and PTSD symptoms by personal characteristics with regression analysis

The logistic regression analysis revealed that men had a lower risk of PTSD symptoms (β = 0.627, CI: 0.39–0.99, *p* = 0.046) than women. The results showed that the risk of PTSD in young people was higher than in the elderly (β = 2.242, CI: 1.03–4.87, *P* = 0.041). The non-healthcare workers had a higher risk of PTSD (β = 2.397, CI: 1.145–5.020, *p* = 0.020) than the healthcare workers. The risk of PTSD was higher in people who did not have enough family income (β = 1.826, CI: 1.01–3.29, *P* = 0.045) or had nearly enough family income (β = 1.59, CI: 1.09–2.32, *P* = 0.014) compared to those who had enough family income. Furthermore, the risk of PTSD was higher in people with underlying disease compared to healthy people (β = 1.647, CI: 1.07–2.54, *P* = 0.024). The people who lived with their family (spouse/children) had a lower risk of PTSD (β = 0.251, CI: 0.08–0.81, *P* = 0.021) than those who lived alone.

There was no significant difference in the risk of depression, anxiety and stress among the age groups. Logistic regression analysis showed that the risk of depression was higher in housewives than in healthcare workers (β = 1.597, CI: 1.04–3.69, *p* = 0.038). In addition, non-healthcare workers had a higher risk of anxiety than healthcare workers (β = 2.158, CI: 1.04–4.47, *p* = 0.038), and the risk of stress in housewives (β = 2.247, CI: 1.17–4.30, *p* = 0.015) and employees (β = 2.761, CI: 1.31–5.83, *p* = 0.008) compared to health care workers.

The risk of depression, anxiety and stress was higher in people who did not have enough family income (β = 1.996, CI: 1.06–3.74, *P* = 0.031; β = 1.975, CI: 1.02–3.80, *P* = 0.042; β = 1.663, CI: 1.13–2.44, *P* = 0.009, respectively) or had nearly enough family income (β = 1.638, CI: 1.13–2.38, *P* = 0.010; β = 1.485, CI: 1.02–2.16, *P* = 0.039, respectively except for stress) compared to those with enough family income.

Furthermore, the risk of anxiety and stress was higher in people with an underlying disease compared to the healthy individuals (β = 1.791, CI: 1.14–2.81, *P* = 0.011; β = 2.376, CI: 1.49–3.76, *P* = 0.000, respectively). Also, the people living with a spouse/family had a lower risk of stress (β = 0.218, CI: 0.05–0.90, *p* = 0.035) than those who lived alone (Table 3).

**Table 3:** Logistic regression analysis of the PTSD symptoms and depression, anxiety, stress with personal characteristics of the participants

Factors		Multiple Logistic Regression															
		PTSD symptoms				Depression				Anxiety				Stress			
		OR	*p*-value	95%CI		OR	*p*-value	95%CI		OR	*p*-value	95%CI		OR	*p*-value	95%CI	
				lower	upper			lower	upper			lower	upper			lower	upper
Gender	female(R)																
	male	0.627	0.046	0.396	0.992	1.138	0.577	0.722	1.794	1.095	0.700	0.691	1.735	0.839	0.451	0.532	1.324
Age	Elderly(R)		0.097				0.945				0.926				0.203		
	young	2.242	0.041	1.033	4.866	0.920	0.834	0.424	2.000	0.955	0.907	0.437	2.087	1.847	0.126	0.841	4.053
	middle-aged	1.734	0.069	0.959	3.135	0.905	0.736	0.507	1.616	1.069	0.823	0.596	1.920	1.651	0.096	0.915	2.981
Occupation	Healthcare (R)		0.212				0.178				0.192				0.054		
	Non-healthcare	2.397	0.020	1.145	5.020	1.915	0.077	0.933	3.931	2.158	0.038	1.042	4.469	2.761	0.008	1.308	5.827
	Teacher/student	1.588	0.158	0.836	3.015	1.470	0.212	0.803	2.688	1.478	0.206	0.807	2.708	1.661	0.104	0.901	3.062
	others	1.522	0.250	0.745	3.109	1.229	0.548	0.627	2.407	1.354	0.383	0.685	2.676	1.888	0.069	0.952	3.743
	Homemaker	1.286	0.460	0.660	2.507	1.957	0.038	1.037	3.693	1.873	0.054	0.988	3.550	2.247	0.015	1.173	4.303
Education Levels	> diploma(R)		0.184				0.692				0.161				0.586		
	Literacy	0.844	0.758	0.289	2.471	1.014	0.978	0.365	2.820	0.510	0.211	0.177	1.466	0.715	0.522	0.257	1.993
	< diploma	2.048	0.060	0.971	4.318	1.218	0.614	0.565	2.626	1.993	0.126	0.823	4.824	1.395	0.412	0.629	3.094
	diploma	1.316	0.288	0.794	2.181	1.356	0.252	0.806	2.283	1.033	0.901	0.617	1.730	1.191	0.512	0.707	2.008
Economic Status	enough(R)		0.022				0.012				0.037				0.033		
	Nearly enough	1.597	0.014	1.099	2.323	1.638	0.010	1.127	2.382	1.485	0.039	1.019	2.163	1.352	0.339	0.729	2.507
	No enough	1.826	0.045	1.013	3.294	1.996	0.031	1.065	3.743	1.975	0.042	1.025	3.805	1.663	0.009	1.133	2.441
Marital Status	married(R)		0.626				0.155				0.774				0.271		
	single	0.638	0.343	0.252	1.616	1.304	0.602	0.481	3.537	1.329	0.564	0.505	3.496	0.632	0.400	0.217	1.840
	divorced	1.260	0.743	0.317	5.011	1.341	0.727	0.257	6.985	1.346	0.688	0.315	5.754	1.914	0.489	0.304	12.031
	died spouse	0.713	0.618	0.189	2.693	0.286	0.105	0.063	1.298	2.262	0.299	0.485	10.551	0.380	0.217	0.082	1.763
Underlying disease	No(R)																
	yes	1.647	0.024	1.070	2.537	1.166	0.486	0.757	1.795	1.791	0.011	1.143	2.807	2.376	0.001	1.499	3.764
Coexistence	single(R)		0.057								0.824				0.292		
	Father/ mother	0.369	0.062	0.130	1.053	0.330	0.103	0.087	1.251	1.172	0.784	0.376	3.655	0.670	0.516	0.201	2.239
	family	0.251	0.021	0.077	0.810	0.221	0.040	0.052	0.934	0.678	0.542	0.195	2.364	0.262	0.057	0.066	1.040
	spouse	0.474	0.226	0.141	1.589	0.185	0.025	0.042	0.807	0.808	0.746	0.222	2.935	0.218	0.035	0.053	0.901
	Childs	0.807	0.709	0.262	2.488	0.629	0.484	0.172	2.302	0.900	0.875	0.242	3.344	1.133	0.854	0.300	4.278
	friends	0.312	0.173	0.058	1.666	0.248	0.137	0.039	1.561	3.154	0.341	0.297	33.487	0.381	0.268	0.069	2.105

*OR* Odds ratio, *R* Reference, *CI* Confidence interval

## Discussion

The study compared the prevalence of depression, anxiety, stress, and PTSD symptoms during the Covid-19 pandemic among three population age groups the elderly, middle-aged and young. Also, the study explored the demographic risk factors of depression, anxiety, stress, and PTSD symptoms during Covid-19 in the study population.

In line with earlier studies, our results recognized a high prevalence of PTSD symptoms (40% with cut-off IES-R > 33), depression (61% with cut-off DASS > 28), anxiety (62% with cut-off DASS > 20), and stress (62% with cut off DASS > 34) in three age groups of the study. In a similar cross-sectional study, in an Australian population of 4126 individuals with a range of 16–82 years, they were assessed in terms of potential psychological distress during the Covid-19 pandemic via DASS-21 and IES-R. The prevalence of moderate-to-severe PTSD symptoms was 43.3%. Also, the rate of moderate-to-severe symptoms in the population was 26.5% for depression, 20.3% for anxiety, and 21.2% for stress [24]. The study revealed that 35.6% of the population of the high-PTSDs group was reported to have an IES-R score above the cut-off [25]. Another longitudinal study among the 1738 Chinese population reported that the average mean IES-R scores of respondents were above the cut-off score [26]. In our results, the rate of depression, anxiety, stress, and PTSD symptoms were high as compared with some studies during the Covid-19 pandemic [24–26]. This may be related to a difference in the study population, socio-cultural differences, and the psychological tools to assess the outcomes.

Our results revealed that the risk of depression, anxiety and stress did not differ among the elderly, middle-aged, and young groups. However, young people were at more risk of PTSD than the elderly. In line with our results, Traunmüller et al. reported that age was not an important factor in determining high PTSD symptoms during the Covid-19 pandemic [24]. A study conducted during this pandemic revealed that the psychological distress level declined at higher ages [27]. Also, a meta-analysis of the Covid-19 impact on public mental health reported that anxiety levels, depression and stress were significantly higher in young than elderly/middle-aged people [7]. The main reason for this seems to be that young people are more concerned about the future consequences, poor economic outcomes, and business closures of the Covid-19 pandemic [28]. Also, larger mental symptoms of young people than in the elderly/middle-aged may be related to their wider access to information through social media, which can also cause psychological distress [29].

Contrary to our hypothesis, elderly people were not at higher risk of psychological symptoms like anxiety, depression, stress, and PTSD symptoms than young people during the pandemic. To explain the finding, the following hypotheses are presented. First, older people have greater resilience to the mental health effects of a crisis [30]. Second, older people tend to underestimate the problems associated with stressful events more than middle-aged and young people. Chen et al. showed that the age difference makes have a different experience of stressful factors types in adulthood. Older adults were less likely than younger adults to use problem-focused coping and reported lower levels of positive affect [31]. Third, a study revealed that coping strategies with stress are related to age. Older people use more adaptive and relaxed coping strategies, like problem-solving, than young/middle-aged people. Also, older people can display greater resilience, lower levels of loneliness, and higher perceived risk and perceived stress, which enables them to limit or avoid exposure to negative experiences. These strategies can prevent the event of a stressor [32].

Of all of the demographic variables that were investigated to be the risk factors for depression (anxiety, stress, and PTSD symptoms), we found that women were at more risk for PTSD than men during the Covid-19 pandemic. Non-healthcare workers were also at higher risk of PTSD symptoms, anxiety and stress than healthcare workers. Similarly, depression and stress risks were higher in homemaker than in healthcare workers. The underlying diseases were a risk factor for PTSD, anxiety and stress. Individuals with low-economic status were at higher risk of PTSD, depression, anxiety and stress than higher-economic-status individuals. Finally, people who were living with their spouse/children had a lower risk of stress and PTSD than those living alone.

In line with our results, some studies recognized that the female gender is a risk factor for PTSD symptoms, as well as anxiety, depression and stress [24, 25]. Previous research confirmed that stressful life events are different for men and women and women experience higher levels of stress for the same events. Therefore, carefully designed interventions are needed in the form of health promotion programs, especially to reduce stress in susceptible people [33]. However, a study reported that after controlling for the covariates, potential risk factors of mental health were similar among males and females [34]. In a longitudinal study in the Netherlands, Vloo et al. investigated gender differences in the mental health impact of the Covid-19 quarantine [35]. The results showed that women experienced more depressive symptoms and disorders, and men experienced anxiety symptoms and disorders caused by the quarantine, each requiring gender-specific policies to improve mental health.

The results of the present study showed that there were fewer mental problems during the Covid-19 crisis in healthcare workers than in non-healthcare workers and homemakers. Most studies reported high levels of mental health problems in healthcare workers during Covid-19 [27–29]. However, scanty research has focused on the comparison between healthcare and non-healthcare workers in this area. A study by Toh et al. (2021) found that healthcare workers reported better mental health than other essential workers and the general population [36]. Also, Schou-Bredal et al. showed that the prevalence of anxiety, depression and PTSD in healthcare workers was lower than in non-healthcare workers [37]. This could be due to several reasons: the assessment method of mental health problems; healthcare system variations across countries; or the infected cases needing hospitalization with or without ventilator treatment.

In line with previous research, our findings confirmed that chronic diseases aggravated the psychological consequences of Covid-19 [34]. A study investigated the impact of Covid-19 on depression and anxiety in patients with chronic medical conditions in Ethiopia. The prevalence of depression and anxiety was reported at 55.7% and 61.8%, respectively. The results emphasized that the history of chronic diseases exacerbated the prevalence of depression and anxiety [38]. Another study reported that a history of regular hospital visits was a significant risk factor for psychological distress in the general population during the Covid-19 pandemic [39]. The high prevalence of psychological distress in people with chronic diseases may be related to the fear of a high mortality rate due to Covid-19 in this group of the population [40].

Nagasu et al. (2021) reported that people with low income perceived more psychological distress than other groups [34]. Another study revealed that people who did not work or had low-average income were significantly at higher risk for depression during the Covid-19 pandemic in China [41]. These findings are consistent with those of the present study. Low-economic status is a potential risk factor for psychological distress even during non-Covid-19 pandemics.

The findings of the present study indicated that people who were living with their spouse/family had a lower risk of stress and PTSD than those living alone. Ozbay et al. believed that social support is necessary for maintaining resilience, and psychological and physical health [42]. It seems that receiving support from the family can prevent physiological distress. Appropriate familial support aids individuals to return to a normal social life after a traumatic event.

Our results highlighted an understanding of the impact of the Covid-19 pandemic on psychological distress among three population ages. The findings may be helpful to mental health professionals to recognize persons who are at a higher risk of developing psychological symptoms and those most in need of interventions during the initial stages of a social crisis. These results may help with the implementation of specific guidelines and protocols to hinder the developing symptoms of depression, anxiety, and PTSD in adults, middle-aged, and elderly populations during a social crisis.

There were some limitations in the present study. Firstly, the method of data collection was an online survey. Thus, the sampling bias might have threatened the results. Secondly, we used self-report scales to assess PTSD symptoms, depression, anxiety, and stress. Further research is suggested to assess psychological disorders with a highly-reliable diagnosis such as clinical intervention. As the elderly participants are not active users of cyberspace, might be technophobic, or could not have internet access, they were not presented in this study. Thirdly, the participants in the online survey might have had different psychological situations during the first and second waves and the period between them. Fourthly, there was a lack of measurement invariance in the Persian versions of the instruments. Therefore, the generalization of the study results to every adult in Iran (people aged 18 and above) should be carried out with caution. Among the research strengths, we can mention population-based sampling, the comparison of three age groups, and accurate data analysis.

## Conclusion

During the Covid-19 pandemic, elderly people are not at higher risk for depression, anxiety, stress, and PTSD symptoms than the young or middle-aged group. Instead, young people were at more risk of psychological symptoms like PTSD than the elderly population. Female gender, non-healthcare workers, chronic diseases, lower economic status, and living alone are risk factors for psychological symptoms during the pandemic. These findings help identification of the populations at risk of mental health problems during Covid-19 and the implementation of national mental health intervention policies in other countries and regions.

## Supplementary Information

**Additional file 1: Fig. 1A.** Normal Q-Q Plot of Depression. **Fig. 1B. **Normal Q-Q Plot of Depression. **Fig. 1C.** Normal Q-Q Plot of Depression. **Fig. 2A.** Normal Q-Q Plot of Anxiety. **Fig. 2B.** Normal Q-Q Plot of Anxiety. **Fig. 2C.** Normal Q-Q Plot of Anxiety. **Fig. 3A. **Normal Q-Q Plot of Stress. **Fig. 3B.** Normal Q-Q Plot of Stress. **Fig. 3C.** Normal Q-Q Plot of Stress. **Fig.4A. **Normal Q-Q Plot ofPTSD total symptoms score. **Fig.4B. **Normal Q-Q Plot ofPTSD total symptoms score. **Fig.4C. **Normal Q-Q Plot ofPTSD total symptoms score. **Fig.5A. **Normal Q-Q Plot ofAvoidance. **Fig.5B.** Normal Q-Q Plot of Avoidance. **Fig.5C.** Normal Q-Q Plot of Avoidance. **Fig.6A.** Normal Q-Q Plot of Intrusion. **Fig.6B.** Normal Q-Q Plot of Intrusion. **Fig.6C.** Normal Q-Q Plot of Intrusion. **Fig.7A.** Normal Q-Q Plot of Hyperarousal. **Fig.7B.** Normal Q-Q Plot of Hyperarousal. **Fig.7C.** Normal Q-Q Plot of Hyperarousal. 

## Data Availability

The datasets generated and/or analyzed during the current study are not publicly available due to ethic issues involving participant’s data and privacy but are available from the corresponding author on reasonable request.

## Abbreviations

DASS-21: Depression, Anxiety and Stress Scale
IES-R: Impact of Event Revised Scale
PTSD: Post-traumatic stress disorder
PTSS: Post-traumatic stress Symptoms
