# Supplementary material for: Depression, anxiety, stress, and PTSD symptoms during the first and second COVID-19 waves: a comparison of elderly, middle-aged, and young people in Iran
Source: BMC Psychiatry. 2023 Mar 23;23:190. doi: 10.1186/s12888-023-04677-0 (PMC10033301; doi:10.1186/s12888-023-04677-0)
Supplement: Supplementary file 1 — Additional file 1: Fig. 1A. Normal Q-Q Plot of Depression. Fig. 1B. Normal Q-Q Plot of Depression. Fig. 1C. Normal Q-Q Plot of Depression. Fig. 2A. Normal Q-Q Plot of Anxiety. Fig. 2B. Normal Q-Q Plot of Anxiety. Fig. 2C. Normal Q-Q Plot of Anxiety. Fig. 3A. Normal Q-Q Plot of Stress. Fig. 3B. Normal Q-Q Plot of Stress. Fig. 3C. Normal Q-Q Plot of Stress. Fig.4A. Normal Q-Q Plot ofPTSD total symptoms score. Fig.4B. Normal Q-Q Plot ofPTSD total symptoms score. Fig.4C. Normal Q-Q Plot ofPTSD total symptoms score. Fig.5A. Normal Q-Q Plot ofAvoidance. Fig.5B. Normal Q-Q Plot of Avoidance. Fig.5C. Normal Q-Q Plot of Avoidance. Fig.6A. Normal Q-Q Plot of Intrusion. Fig.6B. Normal Q-Q Plot of Intrusion. Fig.6C. Normal Q-Q Plot of Intrusion. Fig.7A. Normal Q-Q Plot of Hyperarousal. Fig.7B. Normal Q-Q Plot of Hyperarousal. Fig.7C. Normal Q-Q Plot of Hyperarousal. [file 12888_2023_4677_MOESM1_ESM.docx]

**Supplementary File**

**Normal Q-Q Plots**

**Depression**


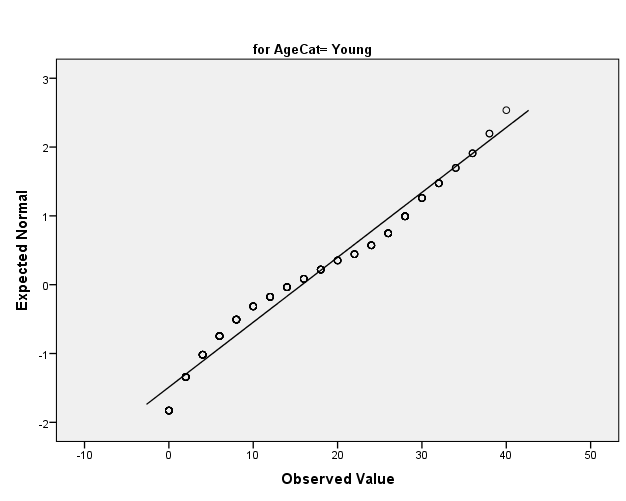


**Fig. 1 A:** Normal Q-Q Plot of Depression


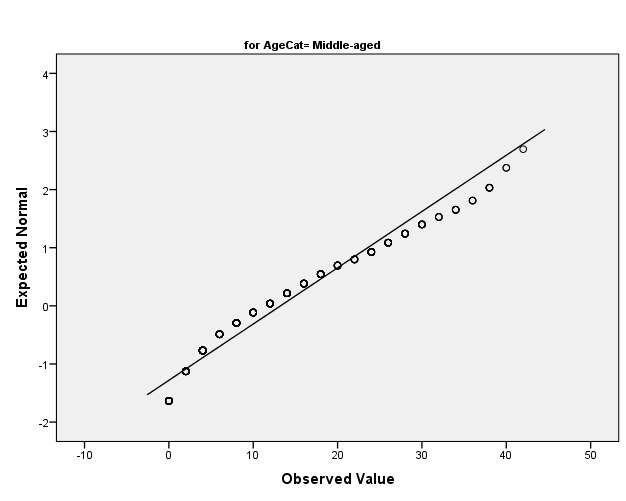


**Fig. 1 B:** Normal Q-Q Plot of Depression


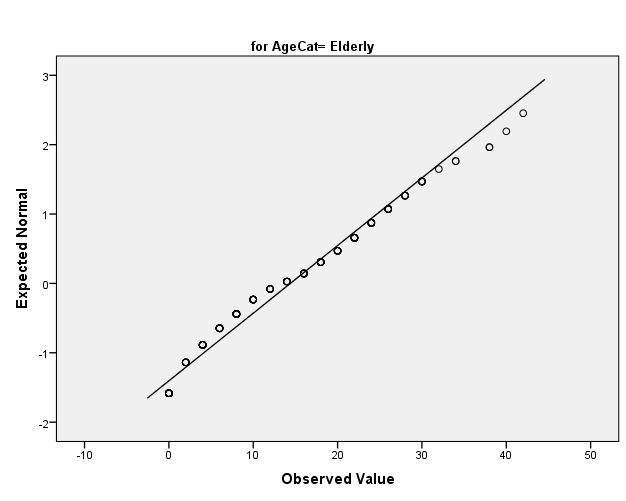


**Fig. 1 C:** Normal Q-Q Plot of Depression

**Anxiety**


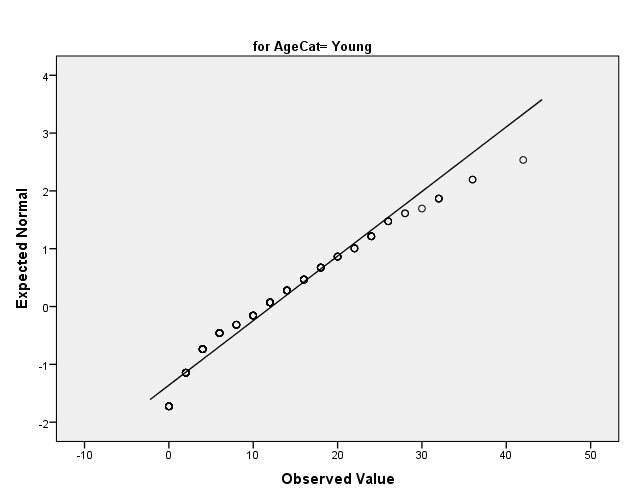


**Fig. 2 A:** Normal Q-Q Plot of Anxiety


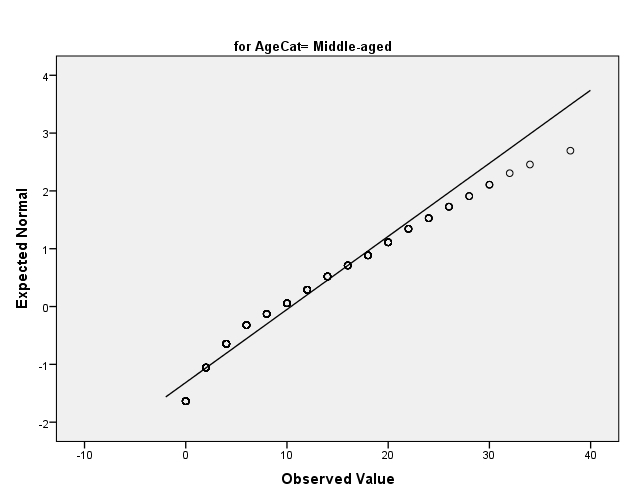


**Fig. 2 B:** Normal Q-Q Plot of Anxiety


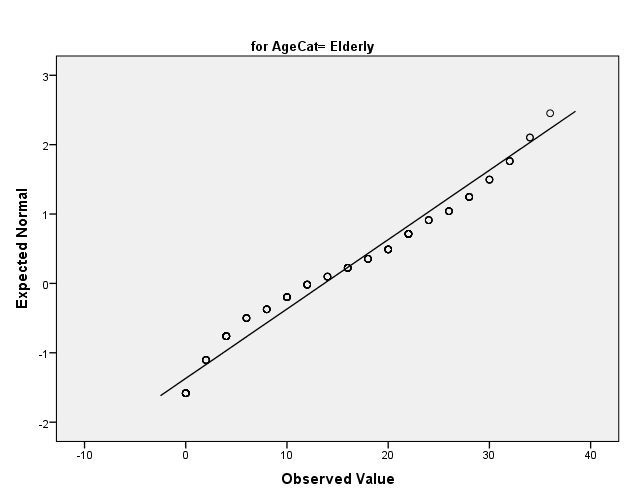


**Fig. 2 C:** Normal Q-Q Plot of Anxiety

**Stress**


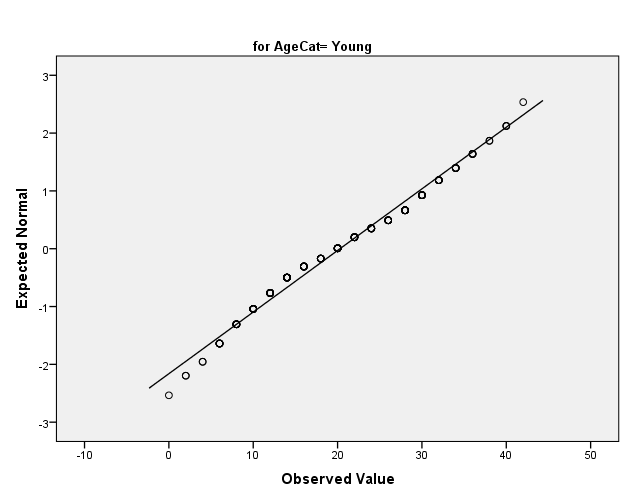


**Fig. 3 A:** Normal Q-Q Plot of Stress


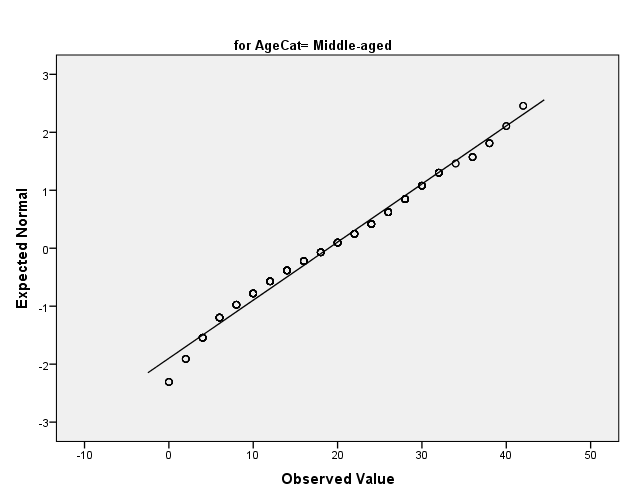


**Fig. 3 B:** Normal Q-Q Plot of Stress


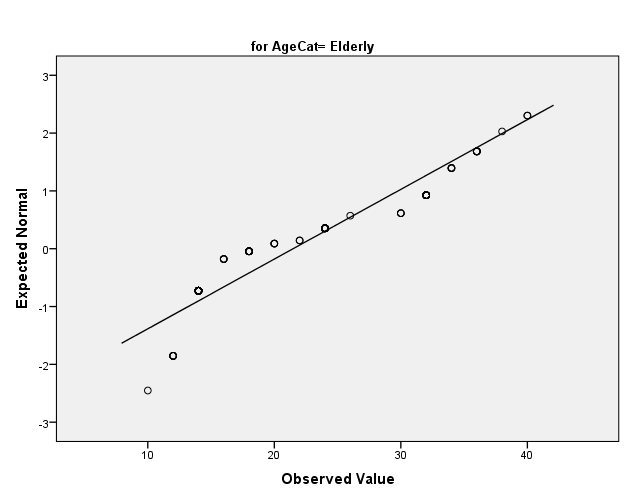


**Fig. 3 C:** Normal Q-Q Plot of Stress

**PTSD total symptoms score**


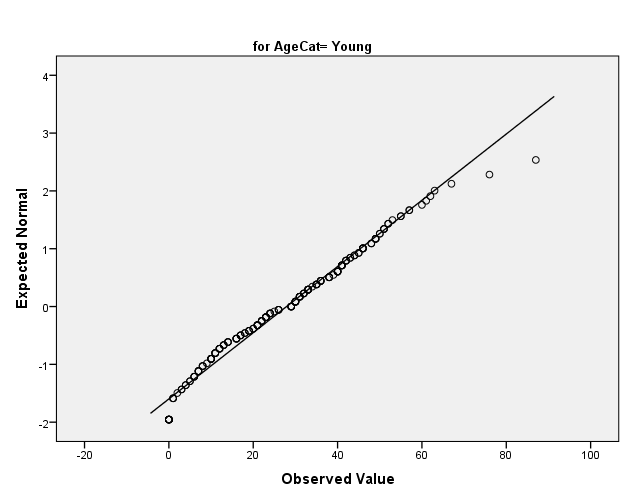


**Fig. 4 A:** Normal Q-Q Plot of PTSD total symptoms score


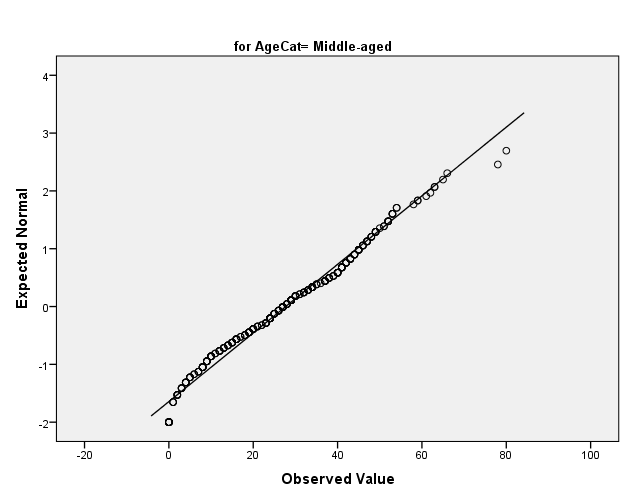


**Fig. 4 B:** Normal Q-Q Plot of PTSD total symptoms score


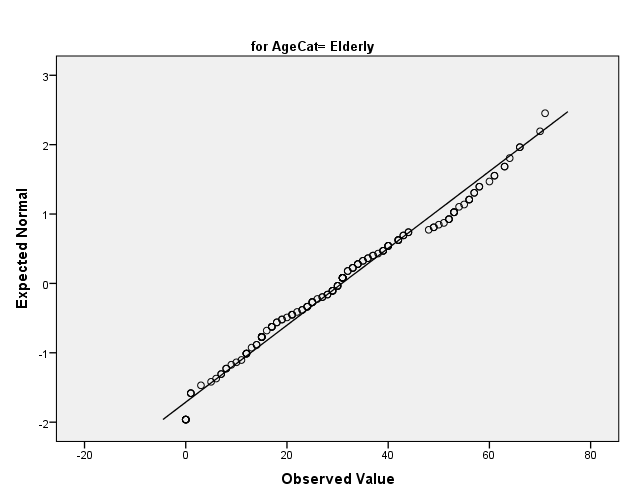


**Fig. 4 C:** Normal Q-Q Plot of PTSD total symptoms score

**PTSD subscales**

**Avoidances**


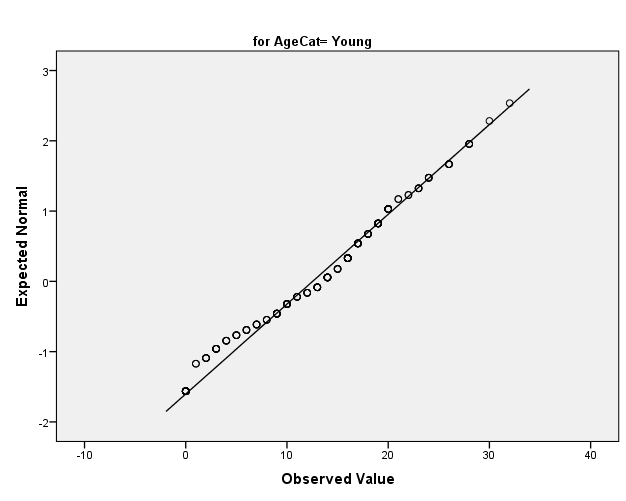


**Fig. 5 A:** Normal Q-Q Plot of Avoidance


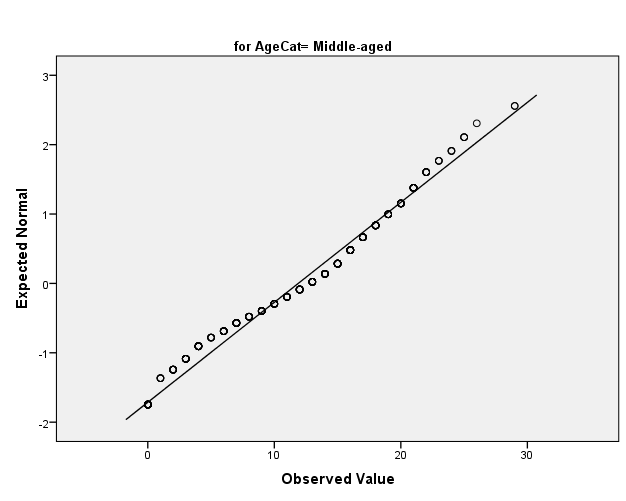


**Fig. 5 B:** Normal Q-Q Plot of Avoidance


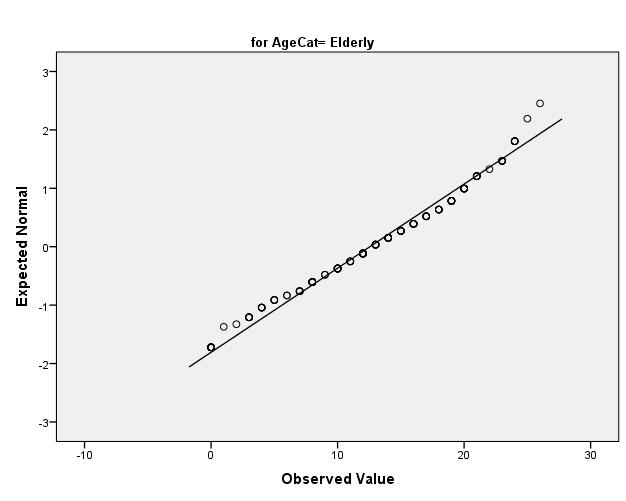


**Fig. 5 C:** Normal Q-Q Plot of Avoidance

**Intrusion**


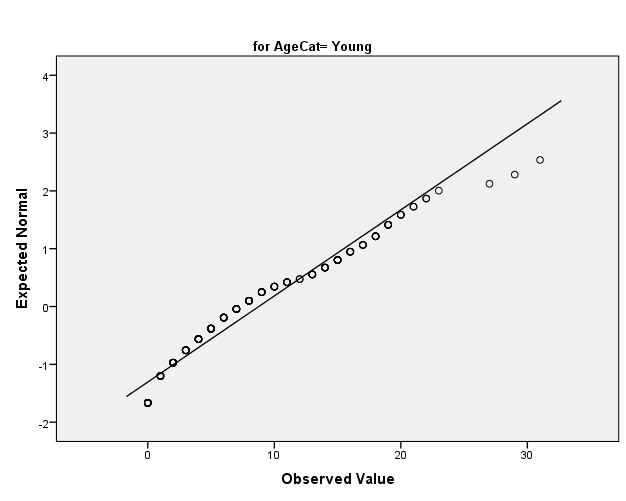


**Fig. 6 A:** Normal Q-Q Plot of Intrusion


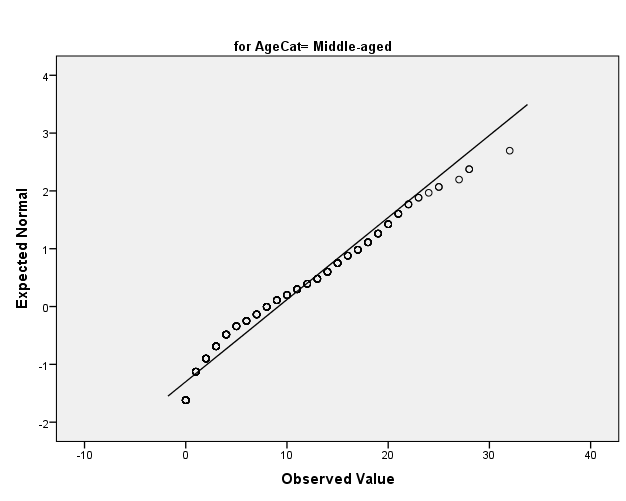


**Fig. 6 B:** Normal Q-Q Plot of Intrusion


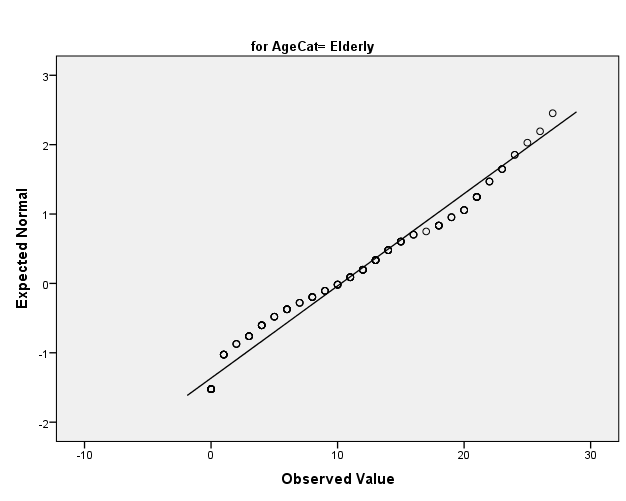


**Fig. 6 C:** Normal Q-Q Plot of Intrusion

**Hyperarousal**


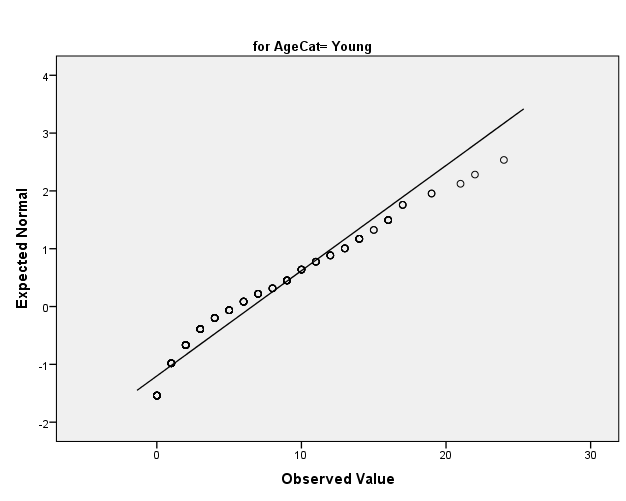


**Fig. 7 A:** Normal Q-Q Plot of Hyperarousal


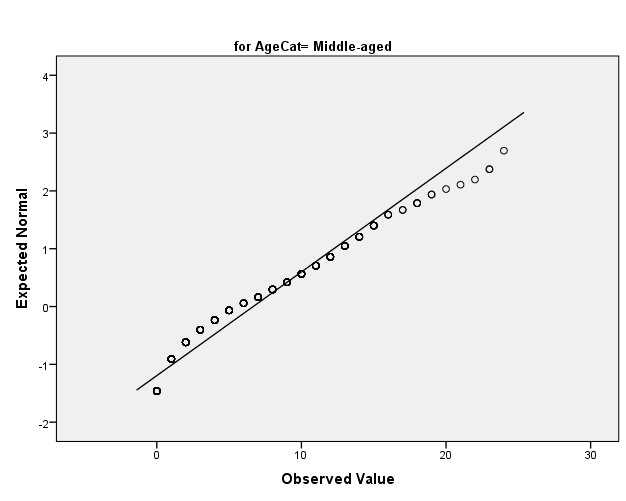


**Fig. 7 B:** Normal Q-Q Plot of Hyperarousal


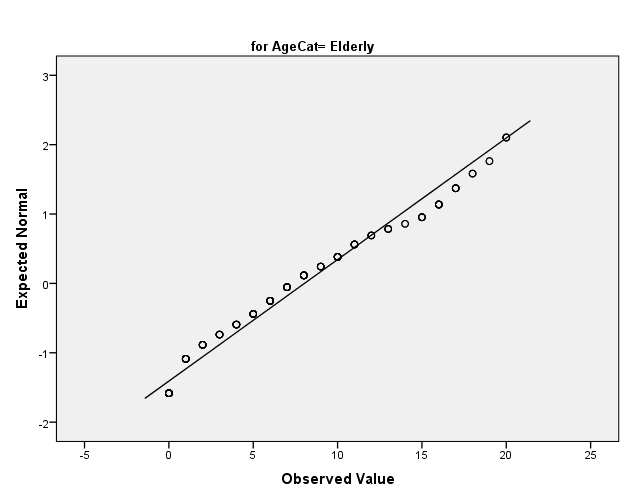


**Fig. 7 C:** Normal Q-Q Plot of Hyperarousal
